# Incidence of respiratory syncytial virus related health care utilization in the United States

**DOI:** 10.7189/jogh.10.020422

**Published:** 2020-12

**Authors:** Sabine Tong, Caroline Amand, Alexia Kieffer, Moe H Kyaw

**Affiliations:** 1 IVIDATA Stats, Levallois-Perret, France; 2 Sanofi, Chilly-Mazarin, France; 3 Sanofi Pasteur, Lyon, France; 4 Sanofi Pasteur, Swiftwater, Pennsylvania, USA

## Abstract

**Background:**

Respiratory Syncytial Virus (RSV) is one of the most frequent causes of acute respiratory infection worldwide. Understanding age-specific health care utilization is necessary to guide effective prevention strategies. This retrospective database analysis assessed the incidence rates of RSV-related health care utilization in the USA over a 7-year period.

**Methods:**

Episodes of RSV were identified in the Truven Health MarketScan^®^ Commercial Claims and Encounters database between 2008 and 2014 using ICD-9-CM codes for pneumonia, bronchiolitis and RSV (480-486, 487.0, 466.1, 491.2, 079.6). Annual RSV-related health care utilization was calculated for the total population, by age group (<1, 1, 2-4, 5-17, 18-49, 50-64, 65-74, 75-84 and ≥85 years) and the proportion of cases for each setting (hospitalization, outpatient, or emergency department [ED] / urgent care [UC]).

**Results:**

Over the 7-year study period, the mean rate of all RSV-associated health care utilization was 2.4 per 1000 person-years, with mean rates ranging from 2.0 to 2.6). The highest rate was seen in infants aged <1 year (mean 79.0 per 1000 over the 7-year period), which decreased with increasing age in the range 2-49 years before increasing with age in older adults (mean rate 8.1 per 1000 over the 7-year period in those ≥85 years). Of all RSV cases, 82% were reported in an outpatient setting, 11% in the ED/UC and 7% were hospitalized.

**Conclusions:**

The annual RSV-related healthcare utilization rates were substantial, especially in infants and young children. These results underscore the need to accelerate the development of RSV prevention strategies to reduce the healthcare burden of RSV.

Respiratory syncytial virus (RSV) is one of the most frequent causes of respiratory tract infections worldwide. It is associated with high reinfection rates, and can lead to more serious symptoms such as pneumonia and bronchiolitis [1]. RSV infections show seasonality, with peaks through the winter months in temperate regions [2,3]. Young children, the elderly, and those with chronic medical conditions are at the greatest risk for severe RSV infections [4,5].

Globally, there were an estimated 33.1 million episodes of RSV-associated respiratory infection in children under 5 years of age, with an estimated 3.2 million requiring hospitalization in 2015 [6]. In children aged <1 year, RSV is believed to be associated with 16 times as many hospitalizations as influenza [7]. In the USA, RSV has been estimated to account for 24% of hospitalizations for lower respiratory tract infections among children aged under 5 years, with hospitalization for RSV highest among those aged <3 months [8]. In those aged <5 years, 1 in every 38 visits to an emergency department (ED) and 1 in every 13 visits to a primary care office each year in the USA were estimated to be related to RSV [9]. RSV is also the leading viral cause of death in children under 5 years in the USA [10,11].

The burden of RSV in the adult population is poorly defined, but is being increasingly recognized as an important cause of morbidity and mortality, particularly in the elderly and adults at high risk – such as those who are immunocompromised, and those with chronic cardiopulmonary conditions. RSV infection has been estimated to occur in 3%-7% of elderly people, and in 4%-10% of high-risk adults [12]; in those over 50 years of age, 7% of RSV-associated infections led to hospitalization [13]. While the highest RSV infection rates are in infants and the young, mortality due to RSV is highest for the elderly in the USA [10,11]. Little is documented regarding RSV in younger adults without comorbid conditions, with one surveillance study identifying 7% of adults age 18-60 years with acute RSV infection, 16% of which were asymptomatic [14], while in adults presenting with a respiratory illness, RSV was identified in 3% [15]. Of adults hospitalized with RSV, 18% were age 20-44 years, while the majority (58%) was 60 years or older [16].

This is the second instalment of analyses assessing the RSV disease burden with a focus on incidence rates associated with health care using the same US database. A previous study assessed the costs and economic burden associated with RSV infection [17]. However, there is still currently limited data describing health care utilization due to RSV across all age groups in the USA. Understanding age-specific health care utilization is necessary to guide effective prevention strategies; this is important for RSV given that there is currently no specific antiviral treatment or vaccination. In this retrospective database study, we assessed the incidence rates of RSV-related health care utilization over a range of age groups in a US population.

## METHODS

### Data source

Patients and their health care utilization were identified from the 2008-2014 Truven Health MarketScan^®^ Commercial Claims and Encounters database and the Medicare database. Together, these databases contain information on an average of 48 982 662 individuals per year from the USA who were insured commercially or as part of the Medicare program during this time period. The Truven Health MarketScan^®^ database is considered representative of the US insured health population with regards to health coverage [18], and includes complete longitudinal records of patient demographics, inpatient services, outpatient services, long-term care, actual paid dollars and net payments by the insurer, and prescription-drug claims covered under a variety of health benefit plans for employees and their dependents from a range of large employers, health plans, and government and public organizations.

All database records are de-identified and fully compliant with US patient confidentiality requirements, including the Health Insurance Portability and Accountability Act (HIPAA) of 1996. As this study used only de-identified patient records, Institutional Review Board (IRB) approval was not required because there was no risk to the subjects and the study did not meet the definition of human subject research.

### Study sample

All enrollees from the MarketScan^®^ enrollment tables were included in order to determine the annual MarketScan^®^ population in person-years from January 1, 2008 to December 31, 2014 for analysis of the annual incidence of RSV-related health care utilization. Persons who were not in the enrollment tables were excluded from analysis. Episodes of RSV between January 1, 2008 and December 31, 2014 were identified; an episode of RSV was defined as an inpatient admission with the principal diagnosis of an RSV-specific or an RSV-attributable respiratory condition (International Classification of Diseases, 9th revision, Clinical Modification code (ICD-9-CM) for all pneumonia, 480-486 (including RSV-specific 480.1); influenza with pneumonia, 487.0; acute bronchiolitis, 466.1 (including RSV specific 466.11); obstructive chronic bronchitis, 491.2; RSV, 079.6) or an outpatient visit with a first or secondary diagnosis of an RSV-specific or RSV-attributable respiratory condition, and a first consultation >28 days after any previous consultation with the same diagnosis code. The first RSV episode occurring during the calendar year was defined as the index episode.

### Outcome measures and definitions

The annual incidence rate of RSV-related health care utilization was defined as the number of RSV cases per 1000 persons based on both RSV-specific and RSV-attributable respiratory conditions. In children <5 years of age it was assumed that 20% of all pneumonia (100% for RSV-specific ICD-9-CM code 4801.1) and influenza with pneumonia diagnoses, and 30% of acute bronchiolitis (100% for RSV-specific ICD-9-CM code 466.11) and obstructive chronic bronchitis diagnoses were caused by RSV, based on previous findings [8]. In those aged ≥5 years, RSV was assumed to attribute to 9% of all pneumonia (100% for RSV-specific ICD-9-CM code 4801.1), influenza with pneumonia, acute bronchiolitis (100% for RSV-specific ICD-9-CM code 466.11) and obstructive chronic bronchitis diagnoses, based on previous findings [12,19].

Demographic data were analyzed for the index RSV episode based on RSV-specific ICD-9-CM codes (079.6, 466.11 or 480.1). These data included age, age group (<1, 1, 2-4, 5-17, 18-49, 50-64, 65-74, 75-84, ≥85 years), sex, US geographic region (Northeast, North Central, South, West, or unknown), and insurance plan type (commercial, Medicare).

The annual proportions of RSV health care utilization were calculated overall and by setting (hospitalization, ED/urgent care (UC) visit, and outpatient visit related to the index visit) using RSV-specific ICD-9-CM codes. If a patient was transferred to several services in the same day for the index event, the most severe service was considered as the setting.

High-risk conditions, categorized based on ICD-9-CM codes for the following conditions: chronic cardiac, pulmonary, renal, metabolic, liver, neurological diseases, diabetes mellitus, hemoglobinopathies, immunosuppressive conditions, and malignancy (Appendix S1 in the **Online Supplementary Document**), were extracted, and computed for RSV cases based on RSV-specific ICD-9-CM codes during each calendar year [20].

### Statistical analyses

For each continuous variable the mean and standard deviation (SD), and median were calculated, and for each categorical variable the frequency and percent were calculated. We determined the incidence rate of all RSV-related health care utilization by dividing the annual number of patients with an RSV episode by the annual number of total enrolled person-years in the MarketScan^®^ databases, and multiplying the result by 1000. The annual incidence rates for each year of the study (2008-2014) were calculated per 1000 person-years with 95% confidence intervals (CI) by the use of a normal approximation. The incidence rates for the entire 7-year study period were calculated as the average of the annual incidence rates during 2008-2014.

Stratified analyses by age groups for the incidence rates of all RSV-related health care utilization and the proportions of high-risk conditions in patients with RSV-specific ICD-9-CM codes were also conducted.

All analyses were performed using SAS^®^ Enterprise Guide 7.1 (SAS Institute, Cary, NC).

## RESULTS

### Patterns of RSV over the entire study period

**Figure 1:**
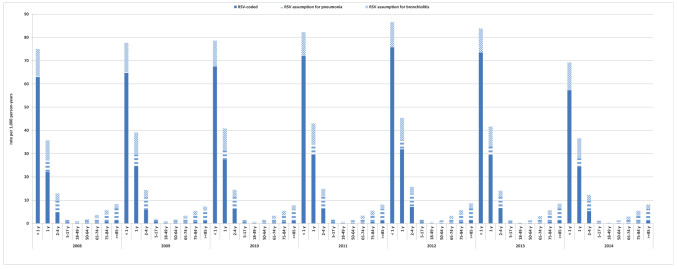
Incidence of respiratory syncytial virus (RSV)-associated disease, 2008-2014. RSV-coded, ICD-9-CM code (079.6, 466.11 or 480.1); RSV assumption for pneumonia, 20% of ICD-9-CM code for all pneumonia (480-486) and influenza with pneumonia (487.0) for <5 years and 9% for ≥5 years; RSV assumption for bronchiolitis 30% of ICD-9-CM code for bronchiolitis (466.1) and obstructive chronic bronchitis (491.2) for <5 years and 9% for ≥5 years.

From 2008 through 2014, on average, there were 41 610 536 annual enrollees in person-years in the Truven Health MarketScan^®^ databases. The average incidence of all RSV-related health care utilization between 2008 and 2014 was 2.4 per 1000 person-years, with mean incidence for each year of the study ranging from 2.0 to 2.6 per 1000 person-years (RSV-specific rate: 1.5 per 1000 person-years [mean rate for individual years ranging from 1.1 to 1.6 per 1000 person-years]) (**Figure 1**).

The annual incidence of RSV-related health care utilization was highest in infants <1 year of age (mean 79.0 per 1000 person-years over the study period), in infants aged 1 year (mean 40.3 per 1000 person-years over the study period), and in children aged 2 to 4 years (mean 14.1 per 1000 person-years over the study period) (**Figure 1** and Table S1 in the **Online Supplementary Document**). In those aged ≥65 years the incidence of RSV-related health care utilization increased with age, with rates for those age ≥85 years two- to three-times higher (mean 8.1 per 1000 person-years over the study period) than in those age 65-74 and 75-84 years (mean 3.3 per 1000 person-years and 5.5 per 1000 person-years over the study period, respectively) (**Figure 1** and **Table S1** in the **Online Supplementary Document**).

The incidence rates of RSV were substantially reduced across several age groups when only RSV-specific ICD-9-CM codes were used; 2- to 3-fold lower in the 2-4 years and 5-17 years age groups, and 5- to 8-fold lower in age groups ≥18 years. The difference between rates of RSV based on RSV-specific ICD-9-CM codes and all RSV-related ICD-9-CM codes were smaller in the <1 year and 1 year age groups (**Figure 1** and Table S1 in the **Online Supplementary Document**).

Of those with RSV-specific diagnosis, slightly more than half (ranging from 53.3% to 54.4% over each study year) of RSV patients were male, with a mean age of 8.9 years (ranging from 7.5 to 10.9 over each study year), many were from the South (ranging from 43.1% to 54.2% over each study year) and North Central (ranging from 18.4% to 22.9% over each study year) regions, and the vast majority were from the commercial claims and encounters database (ranging from 96.5% to 97.1% for each study year) (**Table 1**). Children (0-17 years) represented an average 85% of RSV diagnoses over the whole study period, with the highest proportion in infants <1 year of age (ranging from 43.0% to 48.3% for each study year). Adults (18-64 years) and the elderly (≥65 years) represented only 12% and 3% of cases, respectively over the entire study period.

**Table 1:** Demographic characteristics of patients with respiratory syncytial virus (RSV)-specific ICD-9-CM codes*

Variable, n (%)	2008	2009	2010	2011	2012	2013	2014	2008-2014	
	**(n = 46 457)**	**(n = 58 112)**	**(n = 63 915)**	**(n = 74 858)**	**(n = 76 638)**	**(n = 58 497)**	**(n = 48 812)**	**mean^†^**	
Age (in years), mean (SD)	10.9 (20.8)	10.5 (20.1)	9.6 (19.9)	8.7 (19.3)	7.5 (18.2)	7.7 (18.9)	7.5 (18.4)	8.9	
Age (in years), median	1	1	1	1	1	1	1	1	
**Age (in years), category, n (%):**								
<1	21 024 (45.3)	24 990 (43.0)	28 233 (44.2)	34 196 (45.7)	35 864 (46.8)	28 253 (48.3)	23 221 (47.6)	45.8%	
1	7785 (16.8)	9971 (17.2)	12 005 (18.8)	14 518 (19.4)	15 537 (20.3)	11 552 (19.7)	10 099 (20.7)	19.0%	
2**-**4	5361 (11.5)	7450 (12.8)	8956 (14.0)	10 515 (14.0)	11 334 (14.8)	8411 (14.4)	7026 (14.4)	13.7%	
5**-**17	3063 (6.6)	4762 (8.2)	4076 (6.4)	4563 (6.1)	4371 (5.7)	3045 (5.2)	2580 (5.3)	6.2%	
18**-**49	4774 (10.3)	5723 (9.8)	5142 (8.0)	5183 (6.9)	4433 (5.8)	3031 (5.2)	2408 (4.9)	7.3%	
50**-**64	2982 (6.4)	3499 (6.0)	3498 (5.5)	3494 (4.7)	2885 (3.8)	2168 (3.7)	2028 (4.2)	4.9%	
65**-**74	536 (1.2)	706 (1.2)	831 (1.3)	914 (1.2)	806 (1.1)	748 (1.3)	531 (1.1)	1.2%	
75**-**84	551 (1.2)	628 (1.1)	707 (1.1)	817 (1.1)	730 (1.0)	683 (1.2)	441 (0.9)	1.1%	
≥85	381 (0.8)	383 (0.7)	467 (0.7)	658 (0.9)	678 (0.9)	606 (1.0)	478 (1.0)	0.9%	
Male	24 848 (53.5)	30 957 (53.3)	34 428 (53.9)	40 284 (53.8)	41 674 (54.4)	31 610 (54.0)	26 460 (54.2)	53.9%	
**Insurance:**
Commercial^‡^†	44 968 (96.8)	56 362 (97.0)	61 876 (96.8)	72 438 (96.8)	74 386 (97.1)	56 427 (96.5)	47 327 (97.0)	96.8%	
Medicare§	1489 (3.2)	1750 (3.0)	2039 (3.2)	2420 (3.2)	2252 (2.9)	2070 (3.5)	1485 (3.0)	3.2%	
**US geographic region:**
Northeast	4032 (8.7)	7703 (13.3)	9174 (14.4)	12 376 (16.5)	11 050 (14.4)	8838 (15.1)	8933 (18.3)	14.4%	
North-central	10 367 (22.3)	13 312 (22.9)	13 287 (20.8)	16 367 (21.9)	15 993 (20.9)	11 479 (19.6)	8958 (18.4)	21.0%	
South	25 202 (54.2)	29 771 (51.2)	29 048 (45.4)	32 244 (43.1)	37 582 (49.0)	26 457 (45.2)	23 026 (47.2)	47.9%	
West	6464 (13.9)	7154 (12.3)	11 523 (18.0)	11 681 (15.6)	10 354 (13.5)	10 005 (17.1)	6408 (13.1)	14.8%	
Unknown	392 (0.8)	172 (0.3)	883 (1.4)	2190 (2.9)	1659 (2.2)	1718 (2.9)	1487 (3.0)	1.9%	

*Includes patients with an RSV-specific ICD-9-CM code (079.6, 466.11 or 480.1).

†Means over the entire study period were calculated by dividing the sum of percentages for qualitative variable – means and medians for continuous variable, by the number of years (7).

‡Commercial: CCAE – Active employees and dependent, early (non-Medicare) retirees and dependents, and COBRA continuers.

§Medicare: Retirees (>65 y), their dependents, and younger people with disabilities.

Based on the index RSV event, 7.2% of events were associated with hospitalization, 11.3% were associated with an ED or UC visit, and the most frequent setting for RSV events was the outpatient setting, which represented 81.5% of cases (**Table 2**). Adults and those over 65 years of age tended to be diagnosed with RSV less frequently in the inpatient environment (hospitalization or ED/UC) than children age <5 years. The proportion of index RSV events in the hospital setting appears to be more frequent at the extremes of age, with averages of 9.9% for infants (age <1 year) and 11.8% for those aged ≥85 years.

**Table 2:** Proportion of respiratory syncytial virus (RSV) cases by setting in the United States, 2008-2014*

Age group (years)	Setting, % RSV cases	2008	2009	2010	2011	2012	2013	2014	2008-2014 Mean
**Overall**	Hospitalization	8.0	7.0	7.9	7.2	7.0	6.8	6.4	7.2
	ED/UC visits	9.8	9.6	9.7	11.5	12.0	13.3	13.1	11.3
	Outpatient visits	82.2	83.4	82.5	81.3	81.0	79.9	80.5	81.5
**<1**	Hospitalization	11.6	10.5	11.2	10.0	9.4	8.6	8.0	9.9
	ED/UC visits	12.1	12.2	11.8	13.7	13.7	14.6	14.3	13.2
	Outpatient visits	76.3	77.2	77.0	76.2	76.9	76.8	77.7	76.9
**1**	Hospitalization	8.2	6.3	7.2	5.9	6.0	5.4	4.9	6.3
	ED/UC visits	11.4	12.3	11.5	13.4	13.0	14.2	14.7	12.9
	Outpatient visits	80.5	81.4	81.3	80.7	81.0	80.4	80.5	80.8
**2-4**	Hospitalization	6.9	5.8	6.7	6.0	5.4	5.7	4.7	5.9
	ED/UC visits	10.3	10.4	10.2	12.1	12.3	14.3	15.0	12.1
	Outpatient visits	82.8	83.7	83.0	81.9	82.3	80.0	80.3	82.0
**5-17**	Hospitalization	1.8	1.9	2.4	2.1	2.5	3.3	3.2	2.5
	ED/UC visits	5.2	4.2	4.2	4.6	5.7	6.8	7.1	5.4
	Outpatient visits	93.0	93.9	93.5	93.3	91.8	89.9	89.7	92.2
**18-49 y**	Hospitalization	1.7	2.0	1.7	2.2	2.3	2.6	3.2	2.2
	ED/UC visits	6.6	4.0	4.6	5.6	8.6	12.0	9.0	7.2
	Outpatient visits	91.7	94.0	93.7	92.2	89.1	85.4	87.9	90.6
**50-64**	Hospitalization	1.0	1.3	1.7	1.9	2.1	3.5	4.1	2.2
	ED/UC visits	2.4	1.7	3.1	3.1	4.7	7.4	5.1	3.9
	Outpatient visits	96.6	97.0	95.2	95.0	93.1	89.1	90.8	93.8
**65-74**	Hospitalization	2.6	6.1	5.7	5.0	4.8	6.0	10.2	5.8
	ED/UC visits	2.6	1.0	2.5	3.5	4.5	6.1	4.3	3.5
	Outpatient visits	94.8	92.9	91.8	91.5	90.7	87.8	85.5	90.7
**75-84**	Hospitalization	8.3	6.8	9.3	10.0	8.1	10.2	15.0	9.7
	ED/UC visits	1.3	1.8	1.1	2.9	4.2	5.9	2.7	2.8
	Outpatient visits	90.4	91.4	89.5	87.0	87.7	83.9	82.3	87.5
**≥85**	Hospitalization	10.0	9.1	10.9	14.7	12.1	11.2	14.6	11.8
	ED/UC visits	0.8	0.8	1.3	0.9	1.6	2.3	1.9	1.4
	Outpatient visits	89.2	90.1	87.8	84.3	86.3	86.5	83.5	86.8

ED – emergency department, UC – urgent care

*Includes patients with an RSV-specific ICD-9-CM code (079.6, 466.11 or 480.1)

### Incidence of RSV over each calendar year

**Figure 2:**
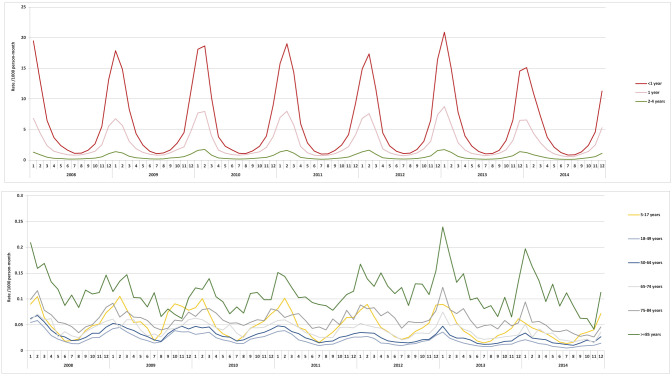
Seasonality of cases of respiratory syncytial virus (RSV) in the United States, 2008-2014. Includes patients with an RSV-specific ICD-9-CM code (079.6, 466.11 or 480.1).

Over the entire study period the number of RSV cases by year varied, with the fewest seen in 2008 (46,457) and the most in 2012 (76,638). The seasonality of RSV was consistent over each of the years of study, with a peak in the winter period; this was consistent across age groups (**Figure 2**). The annual incidence rates of RSV-related health care utilization fluctuated slightly but remained stable throughout the study period among all age groups (Table S1 in the **Online Supplementary Document**). The overall proportions of RSV diagnoses by setting were broadly similar across the years of study (**Table 2**).

### High-risk conditions in patients with RSV

**Figure 3:**
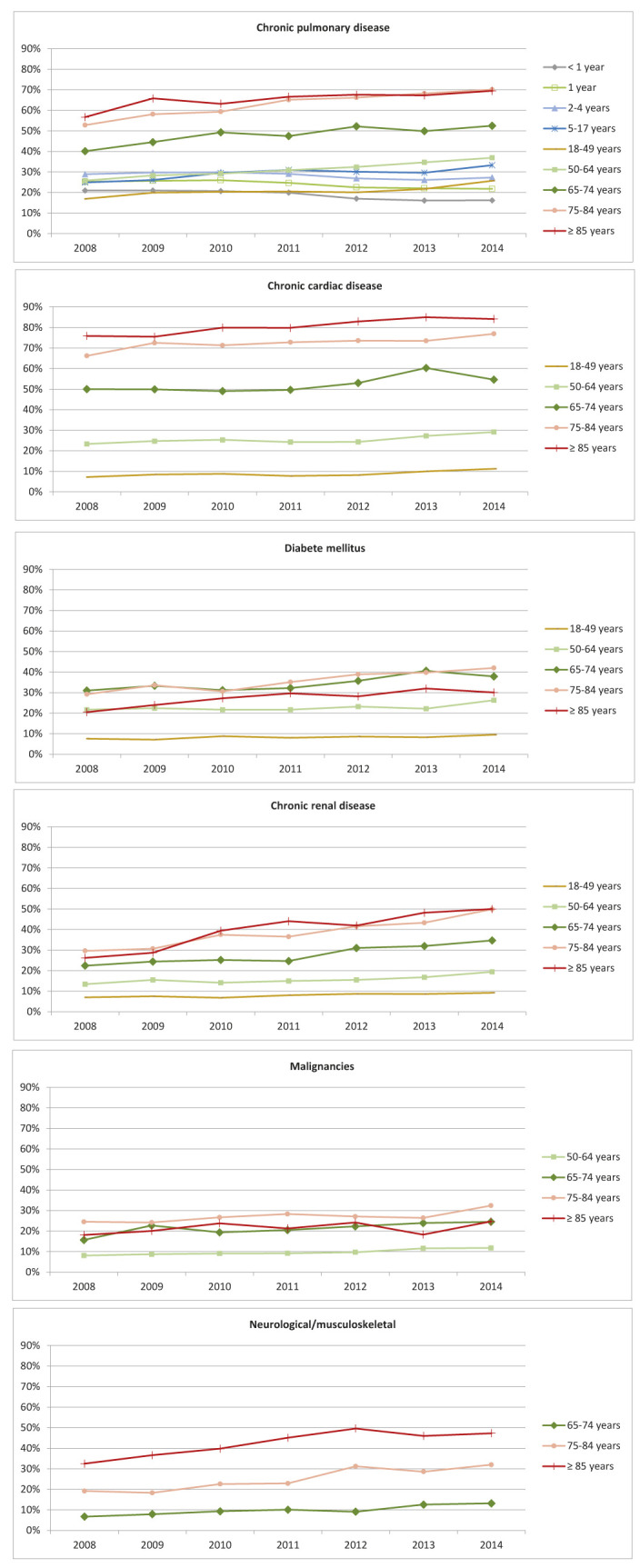
High-risk conditions in RSV patients, 2008-2014. Includes patients with an RSV-specific ICD-9-CM code (079.6, 466.11 or 480.1). Note: Most common high-risk conditions (>20%). Age groups with <10% were not represented in graphs. For conditions and International Classification of Diseases, 9th revision, Clinical Modification (ICD-9-CM) codes for each subgroup of high-risk conditions see Appendix S1 n the **Online Supplementary Document****.**

In patients with RSV, the co-occurrence of high-risk conditions varied by age (**Figure 3**). High-risk conditions were infrequent in children and young adults, and were mainly chronic pulmonary diseases (means ranging from 17%-33% in those with RSV). The occurrence of high-risk conditions in those with RSV increased with age, with older adults and the elderly being most commonly affected by comorbidities such as chronic pulmonary, cardiac and renal diseases, and diabetes. In addition, malignancies and neurological/musculoskeletal diseases were also common in those age ≥75 years. Over time, the proportion of RSV patients also diagnosed with high-risk conditions appears to increase, particularly in those over 65 years of age (**Figure 3**).

## DISCUSSION

These results demonstrate the substantial burden of RSV health care utilization in the USA across all age groups. As has been previously established, the greatest health care utilization for RSV is seen in the youngest populations; in those individuals aged <1 year, utilization was over 95-times that seen in the 18-49 age group. The oldest age groups also exhibited higher rates of RSV-related health care utilization – up to ten-times higher than in the 18-49 age group. As nearly 80% of all RSV-coded infections in the present study were in children <5 years of age, interventions to prevent RSV infection in infants and young children would reduce the substantial burden of RSV related health care utilization in the USA.

Previous studies showed wide variation in reported hospitalizations for RSV [21], possibly due to seasonal variation and differences in study designs (such as case definition, laboratory methods, study populations, study settings/locations, and prospective or retrospective follow-up). The present study showed 6%-8% of patients with RSV were hospitalized for the index episode. The reported rates of hospitalization for RSV vary [3,7-9,15,22,23], which further highlights the challenges in accurately understanding the impact of RSV each year. Despite a lower contribution of RSV hospitalization on overall RSV-related health care utilization when compared to outpatient settings, costs related to RSV hospitalizations accounted for over 60% of total direct health care expenditure for RSV based on economic studies in young children in the USA and Canada [21,24]. Little is known about risk factors that may contribute to a high risk of RSV hospitalization. An improved understanding of the risk factors associated with RSV infections resulting in hospitalization could help identify at-risk groups, and aid the development of preventive measures against severe RSV infections in infants, young children, adults, including those over 65 years of age.

The burden of RSV attributed to infections in the outpatient setting is poorly defined in the USA. Our study included a comprehensive assessment of outpatient health care utilization in various age groups: this assessment showed that 80%-83% of all patients with RSV were seen in the outpatient setting for the index episode. Limited data indicated that RSV in the outpatient setting was estimated to contribute 40% of the health care costs associated with RSV [21], or accounted for a large proportions of RSV disease burden [9,25]. The findings from the current study highlight the substantial burden of RSV outpatient visits in infants and young children (under 2 years old). Compared with RSV hospitalization, the burden of RSV-related outpatient visits were 8-13-times higher in these groups, highlighting the contribution of RSV-related outpatient visits to the total burden of RSV. These data also suggest that the goal for the prevention of RSV should not be restricted to reduction of RSV hospitalization. In addition, we recommend further research on disease dynamics and costs effectiveness models, both to investigate the direct and indirect economic and medical benefits of preventing RSV-related outpatient visits, and to guide effective vaccination polices or other preventive measures.

While the majority of cases of RSV were identified in the outpatient setting, only 11% were associated with an ED/UC visit – this rate is similar to that seen for hospitalization. The proportions in each setting did however vary by age group; compared with the 18-49 age group, the youngest age groups had a greater proportion of cases that were inpatient-related (10% for those <1 year of age) or in the ED/UC setting (13% for those <1 year of age). In the oldest age groups, a greater proportion of cases were also associated with the inpatient setting (10%-12% for age 75 years or older), while ED/UC use was not higher (1%-3%) than for the 18-49 age group. Other studies of RSV-related health care utilization have shown a higher proportion of ED usage in children (approximately 25% were treated in the ED) [9], in those ≥50 years (ED rates twice that of hospitalizations), and in the 18-49 age group (ED rates 6-times higher than hospitalization rates) [15]. Our data reflect previous limited findings which show that very young children with RSV are more likely to present to the ED, as they are at higher risk for severe infection, which is more likely to require UC and hospitalization [4,5]. As with the younger population, older adults are at greater risk for severe RSV [26], and are therefore more likely to require hospitalization.

Patient groups that have been identified as at risk for severe RSV include those who are immunocompromised, and those who have chronic pulmonary or cardiac conditions [4,27]. In our study, those in the oldest age groups and with RSV had a very high co-occurrence of high-risk conditions, in particular chronic pulmonary and chronic cardiac conditions. Other studies of RSV hospitalizations have also shown a high level of comorbid conditions; around a quarter of those admitted in all age groups have been reported to have underlying chronic pulmonary or cardiac conditions [28,29]. In adults aged >50 years, 91% were determined to have comorbid conditions, compared to 64% of those age 18-49 years [15], and it has been shown that 50% of older adults with RSV in the ED had an underlying condition (such as cancer) or were immunocompromised [28]. In addition to the high risk for infection, underlying pulmonary conditions have been shown to be associated with more severe, life-threatening infections [28], and greater mortality in older adults [30]. RSV and influenza in those with chronic lung conditions has also been shown to increase outpatient utilization and prescriptions in all age groups [30]. These data illustrate the particular burden of RSV on those with chronic medical conditions – in particular older adults.

These data support the pattern of seasonality of RSV seen previously [3], with generally consistent levels over the years studied. The further implication of this seasonality is that health care utilization is focused over the winter months, and may also contribute to the excess morbidity and mortality in myocardial infarction, chronic heart failure, COPD, and asthma seen during winter season [11,20,30-32]. Evidence from tracking the seasonality of RSV also suggests that children may be an important source of transmission to older adults, and so tackling RSV in the young may help to reduce incidence in all age groups [3].

Currently, the only available preventative measure against RSV is a monoclonal antibody, palivizumab, which is available for pre-exposure prophylaxis in certain high-risk infants (such as those with congenital heart disease and chronic lung disease) [29]. A Phase 2b study of an RSV monoclonal antibody with an extended half-life (MEDI8897) was conducted by MedImmune/AstraZeneca in healthy preterm infants, and completed in December 2018 (NCT02878330) [33]. As yet, there is no licensed vaccine against RSV, but there are several vaccines currently in development. The most advanced in terms of development is the Novavax RSV F vaccine, which is currently being evaluated in a Phase 3 trial to determine whether vaccination in pregnancy can confer protection for an infant against RSV for the first 90 days of life (NCT02624947) [34], with a Phase 2 trial also ongoing for use in older adults (age 60-80 years) ongoing (NCT03026348) [35]. The introduction of monoclonal antibody prophylaxis or vaccines against RSV will help to substantially reduce the morbidity and mortality related to this infection, particularly in infants, young children, and older adults, who are the most vulnerable to infection [36].

While this analysis uses a large US database to assess RSV-related health care utilization across a range of age groups identifying over 425 000 cases of RSV, there are several limitations that should be considered when interpreting this data. The MarketScan^®^ database includes working-age individuals and their families covered by private insurance supplied by employers who contribute to the database, or Medicare coverage, and is therefore not a random sample of the population as a whole and may not be generalizable to the population of the USA, in particular those who are uninsured. The identification of RSV cases was based on ICD-9-CM coding, which is open to the possibility of misclassification or miscoding as well as missed opportunities for RSV testing. Two approaches were used to identify RSV using ICD-9-CM codes, one which used only RSV-specific codes, which may have led to an underestimation of the number of RSV cases, and a second approach which estimated RSV-attributable respiratory conditions by including a proportion of those with ICD-9-CM codes for pneumonia, influenza with pneumonia, acute bronchiolitis, and obstructive chronic bronchitis in order to try and more accurately reflect the number of RSV cases; this estimation is based on previous reports for the proportion of cases due to RSV [8,12,19], and may introduce an element of error. However, our estimates do not include potential cases of viral wheeze, exacerbation of asthma, acute otitis media or other upper respiratory infections. Therefore, the current study results are likely to underestimate the true burden of RSV infection on health care utilization. This database includes only paid or adjudicated claims, and if an individual did not make an insurance claim, their health care encounter would not have been captured, leading to potential underrepresentation. Additionally, inclusion of historical data regarding the proportion of RSV-related pneumonia, influenza with pneumonia, acute bronchiolitis and obstructive chronic bronchitis diagnoses may affect the estimates and not fully reflect the current incidence. Incomplete information (such as date of birth or clinical outcomes) in the MarketScan^®^ database could also affect our estimates in the study. It is also of note that there was a trend in more recent years for a larger proportion of RSV-cases to occur in high-risk patients, this may be attributable to an increase in the number of people with these chronic conditions [37].

## CONCLUSIONS

These results confirm previous findings of high RSV-related health care utilization in the USA, particularly for the very young and adults aged over 65 years. These data also highlight the very substantial impact of RSV-related outpatient utilization across all age groups. The age-specific data that we present here will help to guide future vaccine policies, and provide critical data for cost-effectiveness models. In addition, these data can be used as a baseline in order to measure the impact of future prevention strategies including monoclonal antibodies or vaccines.

## Additional material

Online Supplementary Document
