# Supplementary material for: Incidence of respiratory syncytial virus related health care utilization in the United States
Source: J Glob Health. 2020 Sep 11;10(2):020422. doi: 10.7189/jogh.10.020422 (PMC7568930; doi:10.7189/jogh.10.020422)
Supplement: Online Supplementary Document [file jogh-10-020422-s001.pdf]

S1 Appendix. High-risk conditions

HIGH RISK CATEGORY	HIGH-RISK SUB-CATEGORY	ICD-9-CM CODES
Chronic cardiac disease	Acute rheumatic fever	391–392
	Chronic rheumatic heart disease	393–398
	Hypertensive heart disease	402, 404
	Ischemic heart disease	410–414
	Diseases of pulmonary circulation	416, 417
	Other forms of heart disease	421, 423, 424, 425, 427.1–427.5, 427.8, 428, 429
	Atherosclerosis, polyarteritis nodosa	440, 446
	Congenital anomalies	745–747
	Surgical/device conditions	V42.1, V45.0, V45.81, V45.82
	Cardiovascular syphilis	093
	Candidal endocarditis	11281
	Myocarditis due to toxoplasmosis	1303
Chronic pulmonary	Other metabolic and immunity disorders	277.0, 277.6
	COPD and allied conditions	491–496
	Pneumoconioses/other lung diseases due to external agents	500–506, 507.0, 507.1, 508
	Other diseases of respiratory system	510, 513–517, 518.0–518.3, 519.0, 519.9
	Congenital anomalies	748.4–748.6, 759.3
	Lung transplant	V426
	Tuberculosis	011, 012
	Diseases due to other mycobacteria	031.0
	Sarcoidosis	135
Chronic renal disease	Hypertensive renal disease	403
	Nephritis, nephrotic syndrome, nephrosis	581–583, 585–587, 588.0, 588.1
	Chronic pyelonephritis	590.0
	Other specified disorders of kidney and ureter	593.8
	Dialysis and transplant	V42.0, V45.1, V56
Diabetes mellitus	Diabetes mellitus	250, 251, 648.0
	Complications of diabetes	357.2, 362.0, 362.11, 366.41
Hemoglobinopathies	Anemias	282–284
Immunosuppressive disorders	HIV/retroviral disease	042–044, 079.5, V08
	Disorders involving immune mechanism	279
	Diseases of blood and blood-forming organs	288.0, 288.1, 288.2

	Polyarteritis nodosa	446
	Diseases of musculoskeletal system and connective tissue	710.0, 710.2, 710.4, 714
	Organ/tissue transplants	V420–V422, V426–V429
	Radiation/chemotherapy	V580, V581
Malignancies		140–208
Other metabolic and immunity disorders	Disorders of adrenal glands	255
	Other disorders	270, 271, 277.2, 277.3, 277.5, 277.8
Liver diseases	Chronic liver disease and cirrhosis	571
	Liver abscess and sequelae of chronic liver disease	572.1–572.8
Neurological/ musculoskeletal	Psychotic conditions	290, 294.1
	Mental retardation	318.1, 318.2
	Hereditary and degenerative diseases of CNS	330, 331, 333.0, 333.4–333.9, 334, 335
	Other disorders of CNS	340, 341, 343, 344.0
	Disorders of peripheral nervous system	358.0, 358.1, 359.1, 359.2
	Late effects of CVD	438
	Chondrodystrophy	756.4

S1 Table. Incidence of RSV-associated disease, 2008–2014

Age group	Rate/1,000 person-years (95% CI)	2008	2009	2010	2011	2012	2013	2014	2008–2014 Average
**Overall**	RSV-associated	2.5 (2.5–2.5)	2.6 (2.5–2.6)	2.4 (2.4–2.4)	2.5 (2.5–2.5)	2.5 (2.5–2.5)	2.3 (2.3–2.4)	2.0 (2.0–2.0)	2.4
	RSV-coded	1.5 (1.5–1.5)	1.6 (1.5–1.6)	1.5 (1.5–1.5)	1.5 (1.5–1.6)	1.6 (1.6–1.6)	1.4 (1.4–1.5)	1.1 (1.1–1.1)	1.5
	RSV assumption for pneumonia	0.8 (0.8–0.8)	0.8 (0.8–0.8)	0.7 (0.7–0.7)	0.8 (0.8–0.8)	0.8 (0.8–0.8)	0.7 (0.7–0.7)	0.7 (0.7–0.7)	0.8
	RSV assumption for bronchiolitis	0.2 (0.2–0.2)	0.2 (0.2–0.2)	0.2 (0.2–0.2)	0.2 (0.2–0.2)	0.1 (0.1–0.1)	0.2 (0.2–0.2)	0.2 (0.2–0.2)	0.2
**<1 year**	RSV-associated	75.0 (74.1–75.9)	77.6 (76.7–78.5)	78.6 (77.7–79.4)	82.2 (81.4–83.0)	86.6 (85.7–87.4)	83.8 (82.9–84.7)	69.2 (68.4–70)	79.0
	RSV-coded	63.0 (62.1–63.8)	64.7 (63.9–65.5)	67.5 (66.7–68.3)	72.0 (71.2–72.8)	75.7 (74.9–76.5)	73.5 (72.6–74.3)	57.2 (56.5–57.9)	67.6
	RSV assumption for pneumonia	0.4 (0.3–0.4)	0.3 (0.3–0.4)	0.0 (0.0–0.0)	0.0 (0.0–0.0)	0.0 (0.0–0.0)	0.0 (0.0–0.0)	0.1 (0.1–0.2)	0.1
	RSV assumption for bronchiolitis	11.7 (11.3–12.1)	12.5 (12.2–12.9)	11.1 (10.8–11.4)	10.2 (9.9–10.5)	10.8 (10.5–11.1)	10.3 (10.0–10.6)	11.9 (11.5–12.2)	11.2
**1 year**	RSV-associated	35.7 (35.1–36.3)	39.1 (38.5–39.7)	40.8 (40.2–41.4)	43.0 (42.4–43.6)	45.4 (44.8–46.0)	41.6 (41.0–42.2)	36.6 (36.0–37.1)	40.3
	RSV-coded	22.2 (21.7–22.6)	24.6 (24.1–25.1)	27.3 (26.8–27.7)	29.6 (29.2–30.1)	31.9 (31.4–32.4)	29.4 (28.8–29.9)	24.3 (23.8–24.8)	27.0
	RSV assumption for pneumonia	4.9 (4.6–5.1)	4.9 (4.7–5.1)	4.3 (4.1–4.5)	4.1 (4.0–4.3)	3.9 (3.8–4.1)	3.6 (3.5–3.8)	3.6 (3.4–3.8)	4.2
	RSV assumption for bronchiolitis	8.7 (8.4–9.0)	9.7 (9.4–10.0)	9.3 (9.0–9.6)	9.2 (9.0–9.5)	9.5 (9.3–9.8)	8.6 (8.3–8.9)	8.6 (8.3–8.9)	9.1
**2–4 years**	RSV-associated	12.9 (12.7–13.1)	14.3 (14.1–14.5)	14.4 (14.2–14.6)	14.8 (14.7–15.0)	15.7 (15.5–15.9)	14.1 (13.9–14.3)	12.2 (12.0–12.4)	14.1
	RSV-coded	4.8 (4.7–5.0)	5.8 (5.6–5.9)	6.2 (6.1–6.4)	6.5 (6.3–6.6)	7.1 (7.0–7.2)	6.6 (6.5–6.7)	5.3 (5.2–5.4)	6.0
	RSV assumption for pneumonia	5.1 (5.0–5.2)	5.4 (5.2–5.5)	5.1 (4.9–5.2)	5.3 (5.2–5.4)	5.4 (5.2–5.5)	4.7 (4.6–4.8)	4.2 (4.1–4.3)	5.0
	RSV assumption for bronchiolitis	3.0 (2.9–3.1)	3.2 (3.1–3.3)	3.1 (3.0–3.2)	3.1 (3.0–3.2)	3.3 (3.2–3.3)	2.8 (2.7–2.9)	2.7 (2.6–2.8)	3.0
**5–17 years**	RSV-associated	1.5 (1.5–1.5)	1.8 (1.8–1.8)	1.4 (1.4–1.5)	1.6 (1.6–1.7)	1.6 (1.6–1.6)	1.3 (1.3–1.3)	1.2 (1.1–1.2)	1.5
	RSV-coded	0.5 (0.5–0.6)	0.7 (0.7–0.7)	0.5 (0.5–0.6)	0.5 (0.5–0.6)	0.5 (0.5–0.5)	0.4 (0.4–0.5)	0.4 (0.3–0.4)	0.5
	RSV assumption for pneumonia	0.8 (0.8–0.8)	0.9 (0.9–0.9)	0.8 (0.8–0.8)	1.0 (0.9–1.0)	0.9 (0.9–1.0)	0.7 (0.7–0.8)	0.7 (0.7–0.7)	0.8
	RSV assumption for bronchiolitis	0.1 (0.1–0.2)	0.2 (0.2–0.2)	0.1 (0.1–0.1)	0.1 (0.1–0.1)	0.1 (0.1–0.1)	0.1 (0.1–0.1)	0.1 (0.1–0.1)	0.1
**18–49 years**	RSV-associated	0.9 (0.9–1.0)	0.9 (0.9–0.9)	0.8 (0.8–0.8)	0.8 (0.8–0.8)	0.7 (0.7–0.7)	0.6 (0.6–0.6)	0.6 (0.6–0.6)	0.8
	RSV-coded	0.3 (0.3–0.3)	0.3 (0.3–0.4)	0.3 (0.3–0.3)	0.2 (0.2–0.2)	0.2 (0.2–0.2)	0.2 (0.2–0.2)	0.1 (0.1–0.1)	0.2
	RSV assumption for pneumonia	0.5 (0.5–0.5)	0.5 (0.5–0.5)	0.4 (0.4–0.4)	0.4 (0.4–0.4)	0.4 (0.4–0.4)	0.4 (0.4–0.4)	0.4 (0.4–0.4)	0.4
	RSV assumption for bronchiolitis	0.1 (0.1–0.1)	0.1 (0.1–0.1)	0.1 (0.1–0.1)	0.1 (0.1–0.1)	0.1 (0.1–0.1)	0.1 (0.1–0.1)	0.1 (0.1–0.1)	0.1

**50–64 years**	RSV-associated	1.8 (1.7–1.8)	1.6 (1.6–1.7)	1.5 (1.5–1.5)	1.5 (1.5–1.5)	1.4 (1.4–1.4)	1.4 (1.4–1.4)	1.3 (1.3–1.4)	1.5
	RSV-coded	0.4 (0.4–0.4)	0.4 (0.4–0.4)	0.3 (0.3–0.4)	0.3 (0.3–0.3)	0.2 (0.2–0.3)	0.2 (0.2–0.2)	0.2 (0.2–0.2)	0.3
	RSV assumption for pneumonia	0.9 (0.9–0.9)	0.8 (0.8–0.8)	0.7 (0.7–0.8)	0.8 (0.8–0.8)	0.8 (0.8–0.8)	0.8 (0.8–0.8)	0.7 (0.7–0.8)	0.8
	RSV assumption for bronchiolitis	0.4 (0.4–0.5)	0.4 (0.4–0.4)	0.4 (0.4–0.4)	0.4 (0.4–0.4)	0.4 (0.4–0.4)	0.4 (0.4–0.4)	0.4 (0.4–0.4)	0.4
**65–74 years**	RSV-associated	3.6 (3.5–3.7)	3.3 (3.2–3.4)	3.3 (3.2–3.4)	3.3 (3.2–3.4)	3.2 (3.2–3.3)	3.1 (3.1–3.2)	2.9 (2.9–3.0)	3.3
	RSV-coded	0.5 (0.4–0.5)	0.5 (0.4–0.5)	0.5 (0.4–0.5)	0.4 (0.4–0.4)	0.4 (0.4–0.4)	0.4 (0.3–0.4)	0.3 (0.2–0.3)	0.4
	RSV assumption for pneumonia	1.8 (1.7–1.8)	1.6 (1.5–1.6)	1.5 (1.5–1.6)	1.6 (1.5–1.6)	1.6 (1.5–1.6)	1.5 (1.5–1.6)	1.5 (1.4–1.5)	1.6
	RSV assumption for bronchiolitis	1.3 (1.3–1.4)	1.3 (1.2–1.3)	1.3 (1.2–1.3)	1.3 (1.3–1.4)	1.3 (1.2–1.3)	1.3 (1.2–1.3)	1.2 (1.2–1.3)	1.3
**75–84 years**	RSV-associated	5.7 (5.5–5.8)	5.4 (5.2–5.5)	5.4 (5.3–5.6)	5.5 (5.4–5.6)	5.7 (5.6–5.8)	5.6 (5.5–5.8)	5.4 (5.2–5.5)	5.5
	RSV-coded	0.7 (0.6–0.7)	0.6 (0.6–0.7)	0.6 (0.6–0.7)	0.6 (0.6–0.6)	0.6 (0.6–0.7)	0.6 (0.6–0.7)	0.4 (0.4–0.5)	0.6
	RSV assumption for pneumonia	3.1 (3.0–3.2)	2.8 (2.7–3.0)	2.8 (2.7–2.9)	2.9 (2.8–3.0)	3.0 (2.9–3.1)	2.9 (2.8–3.0)	2.9 (2.8–3.0)	2.9
	RSV assumption for bronchiolitis	1.9 (1.8–2.0)	1.9 (1.8–2.0)	2.0 (1.9–2.1)	2.0 (1.9–2.1)	2.1 (2.0–2.2)	2.1 (2.0–2.2)	2.1 (2.0–2.2)	2.0
**≥85 years**	RSV-associated	8.3 (8.0–8.7)	7.2 (6.9–7.5)	7.8 (7.5–8.0)	8.1 (7.8–8.3)	8.7 (8.4–8.9)	8.5 (8.2–8.7)	8.1 (7.9–8.4)	8.1
	RSV-coded	1.3 (1.2–1.5)	1.0 (0.9–1.1)	1.1 (1.0–1.2)	1.2 (1.1–1.2)	1.3 (1.2–1.4)	1.3 (1.2–1.4)	1.1 (1.0–1.2)	1.2
	RSV assumption for pneumonia	5.2 (4.9–5.5)	4.5 (4.3–4.7)	4.8 (4.6–5.0)	5.0 (4.8–5.2)	5.2 (5.0–5.4)	5.1 (4.9–5.3)	5.0 (4.8–5.2)	5.0
	RSV assumption for bronchiolitis	1.8 (1.6–1.9)	1.7 (1.6–1.8)	1.9 (1.7–2.0)	2.0 (1.8–2.1)	2.1 (2.0–2.2)	2.1 (1.9–2.2)	2.0 (1.9–2.2)	1.9

RSV-coded, ICD-9-CM code (079.6, 466.11 or 480.1); RSV assumption for pneumonia, 20% of ICD-9-CM code for all pneumonia (480–486) and influenza with pneumonia (487.0) for <5 years and 9% for ≥5 years; RSV assumption for bronchiolitis 30% of ICD-9-CM code for bronchiolitis (466.1] and obstructive chronic bronchitis (491.2) for <5 years and 9% for ≥5 years.
